# Successful pregnancy and live birth in woman with congenital adrenal hyperplasia: A case report

**DOI:** 10.1097/MD.0000000000023495

**Published:** 2020-12-11

**Authors:** Wei Xiong, Guiqiong Huang, Xiaodong Wang, Shiqiao Tan

**Affiliations:** Department of Gynecology and Obstetrics, West China Second University Hospital, Sichuan University, Key Laboratory of Birth Defects and Related Diseases of Women and Children (Sichuan University), Ministry of Education, Chengdu, Sichuan, P.R. China.

**Keywords:** 21-hydroxylase deficiency, congenital adrenal hyperplasia, glucocorticoid, successful pregnancy

## Abstract

**Rationale:**

Women with congenital adrenal hyperplasia (CAH) can suffer from impaired fertility rates as a result of increased androgen secretion or impaired sex steroid production. CAH patients have lower pregnancy rate compared to normal women. Only a few cases with successful pregnancy have been reported in the literature. This report described a case of CAH with successful pregnancy and live birth.

**Patient concerns:**

A 23-year-old woman visited our endocrinology department for clitoral hypertrophy and primary amenorrhea.

**Diagnoses:**

The patient was diagnosed as CAH.

**Intervention:**

Prednisone was initially started to improve the patient's symptoms. Then she underwent clitoral resection and vaginoplasty several months later. She continuously took the prednisolone after the operation and had been undergoing regular checkups.

**Outcomes:**

She was pregnant spontaneously without assisted reproductive technology and had a successful live birth. Her baby had shown normal external genitalia with normal karyotype and normal development up to 6 years of age.

**Lessons:**

Some mild CAH patients with certain types can achieved successful pregnancy without any assisted reproductive technology after treatment with steroid. The pregnancy rate among CAH women who wish to conceive may be much more optimistic than previous researches.

## 1 Introduction

Congenital adrenal hyperplasia (CAH) refers to a general term for a group of monogenic autosomal recessive disorders involving enzymes deficiencies in the transformation of cholesterol to cortisol within the adrenal cortex.^[1]^ Deficiency of 21-hydroxylase is the most common cause leading to CAH which has been distributed into 2 forms: classical and non-classical (NCCAH) according to the degree of damage to 21-hydroxylase activity.^[2]^ The classical form is further separated into the salt-wasting (SW) and simple-virilizing (SV).^[2]^ The diagnosis of CAH is built on clinical symptoms (such as atypical genitalia to variable degrees, hirsutism, oligomenorrhea, and infertility) in females, testing for serum 17-hydroxyprogesterone, and genetic testing. Almost all classic CAH patients require glucocorticoid treatment (often with mineralocorticoid), while in the milder NCCAH, treatment is given when patients get symptoms due to hyperandrogenemia.

Compared to the general population, adult women with CAH have a higher occurrence of spontaneous miscarriage and lower fertility rates.^[3]^ The possibility of spontaneous pregnancy and infertility is correlated with the severity of the enzyme deficiency.^[4]^ Here, we report a case of a woman with CAH who was treated with glucocorticoids and had successful pregnancy with live birth.

## 2 Case report

A 23-year-old woman first visited our department for clitoral hypertrophy and primary amenorrhea. Clitoral hypertrophy without dysuria was found at the age of 5 years old. She had no breast development and menarche until visitation and the patient did not consider this unusual. She had accelerated body growth from when she was 8 years old but the growth was arrested at the age of 12. Transvaginal ultrasound in our hospital revealed “immature uterus, inferior vaginal atresia” and the result of chromosomal study showed a normal karyotype (46, XX). Hormonal assay revealed elevated adrenocorticotropic Hormone (ACTH), the high concentration of progesterone and 17-hydroxy progesterone, which accorded with 21-hydroxylase deficiency. She took prednisone (maximum dose 15–minimum 7.5 mg QD) after visitation. One month later, her breasts slightly expanded and menstruation began with alleviative hirsutism, slightly diminished clitoris. The treatment with prednisone 7.5 mg QD was sustained without any complication. Since then, menstruation had been regular every month, with periods of 5 to 8 days and cycles of 25 to 28 days. Normal ACTH and testosterone were noted in the third month after medication.

She underwent clitoral resection and vaginoplasty at our department 5 months later. During the operation, we found her clitoris resembled a small penis with a diameter of 1.5 cm, clitoral head of about 0.8 cm and a clitoral body length of about 5 cm. The perineal body covered the vagina and urethral orifice. After its separation, the vaginal orifice (about 0.5 cm wide), thin urogenital septum and urethral orifice could be seen. The length of the vagina was 8 cm with the probe. We revised the middle of the perineum longitudinally for about 4 cm, and then sutured to form the vaginal orifice that could accommodate a finger. She continuously took the prednisolone (mini-mum dose of 5 mg/d to a maximum dose of 10 mg/d) after the operation and had been undergoing regular checkups at the endocrinology department.

She was married at 27 years old and pregnant spontaneously soon without any assisted reproductive technology. She had her entire prenatal examination at another hospital. She was diagnosed with gestational diabetes mellitus (GDM) in the oral glucose tolerance test conducted at 25+2 weeks of gestation. Blood sugar was well-controlled by lifestyle modification recommended by clinical dietitian without any medication. The development of a fetus was according to the gestational age without any sign of congenital malformation. She continued to take prednisolone 10 mg/d during pregnancy advised by an endocrinologist. Cesarean section was conducted rather than vaginal delivery because of the history of vaginoplasty. A healthy female baby who weighed 3420 g with 10–10–10 Apgar score was delivered by elective cesarean section at 39 weeks of gestation. After the delivery, the patient had taken prednisolone (7.5 mg/d) consistently for the CAH. The 6-years-old child has shown normal external genitalia with normal karyotype (46, XX) and normal development up to now.

## 3 Disscusion

CAH is characterized by a deficiency in one of the enzymes or proteins involved in cortisol biosynthesis. The forms of CAH in current researches include 21-hydroxylase deficiency, congenital lipoid adrenal hyperplasia (steroidogenic acute regulatory protein), deficiencies of 11β-hydroxylase, 3β-hydroxysteroid dehydrogenase or 17α-hydroxylase/17, 20-lyase, and cytochrome P450 oxidoreductase deficiency.^[4]^ Decreased cortisol production scales up ACTH levels, chronic adrenal stimulation, and consequently, a characteristic combination of elevated steroid precursors and deficient products that are distinctive for each form of CAH.^[4]^ It is a known fact that the most common form, 21-hydroxylase deficiency, represents more than 90% of all cases.^[5]^

SW CAH, caused by the severe impairment of the 21-hydroxylase enzyme (< 2% enzymatic activity), leads to hypovolemia, hyponatremia, hyperkalemia, hyperreninemia, failure to thrive, seizure, and ultimately death in a newborn due to inadequate production of aldosterone. Therefore, early diagnosis and appropriate cure should be conducted immediately after birth.^[5]^ Compared to SW CAH, SV CAH has an increase of about 1% to 2% of enzyme 21-hydroxylase activity, leading to overproduction of adrenal androgens but adequate aldosterone. Androgens are high and females are also born with variable degrees of genital virilization in SV CAH. NCCAH refers to 20 to 50% normal function of 21 hydroxylase enzymes. In children, Clinical signs of NCCAH may include premature pubarche, premature adrenarche, cystic acne, accelerated growth, advanced bone age, and short final height.^[6–9]^ Later in life, hirsutism (60%–78%), menstrual cycle disorders (55%), acne (33%) and decreased fertility (12%) are frequent presenting features.^[10,11]^

Early diagnosis and the treatment are vital for classic CAH to get rid of the cortisol deficiency, suppress ACTH overproduction and effects of hyperandrogenemia, and to correct external genitalia. Almost all patients with classic CAH require long-lasting glucocorticoid replacement treatment, and mineralocorticoid replacement is required as well in SW CAH requires.^[4]^ In children, the combined treatment with growth hormone or a LHRH analog can notably improve the patient's final adult height.^[12]^ In our case, prednisolone was initially taken at the age of 23 years old. Because of their negligence, she missed the best opportunity for treatment to improve the final adult height. In NCCAH patients, glucocorticoid alone with a small dose at bedtime is sufficient to suppress the effects of androgen. In NCCAH women with good adrenal suppression, an androgen antagonist may be a useful adjuvant therapy to improve androgenic symptoms, and oral contraceptives may be a beneficial add-on therapy for concomitant polycystic ovary syndrome.^[2]^

Previous studies showed a low fertility rate for female patients with classic CAH, especially among those with the SW form. Fertility in patients with NCCAH appears to be mildly reduced. A recent multicenter, cross-sectional study in Europe found that 15% of all patients with 46, XX CAH (28 of 221) had conceived naturally, and 2% (4 of 221) conceived with the aid of assisted reproductive technology.^[13]^ Our patient was pregnant spontaneously without any assisted reproductive technology. Chronic anovulation and endometrial dysfunction have been suggested to explain this low delivery rate in female patients with 21 hydroxylase deficiency.^[14,15]^ The most obvious factor that can lead to these abnormalities in CAH is excess adrenal androgens, including adrenal hypersecretion of progesterone through altering normal central feedback pathways, interfering with the gonadotropin-releasing hormone pulse generator and negatively affecting reproductive function.^[4,16]^ Moreover, it has been suggested that elevated serum androgens can affect folliculogenesis directly and modulate ovarian hormone secretion by several pathways.^[4]^ Knobil E, et al reported that estrogens generated from excess androgens can suppress the (hypothalamic-pituitary-gonadal axis and thus lead to anovulation and irregular menstrual cycles.^[17]^ Meanwhile, excess progesterone concentrations may alter the gonadotropin-releasing hormone pulse generator, disrupt endometrial development, make the cervical mucus thicker, and adversely affect the quality of oocytes and so on.^[4]^

Women with classic CAH were reported being less sexually active and engaging in relationships less frequently than the general population.^[18]^ There have been studies that postsurgical difficulties, psychosexual development, and psychological factors may play a role in the reduced pregnancy rates in women with classic CAH.^[4]^ Despite these issues, the pregnancy rate among CAH women who wish to conceive is much more optimistic than previous researches. A study showed women in classic CAH group had a 91% pregnancy rate, which was the same as the rate found in the normal control population (95%), similar in the SV and SW subgroups (93% and 89%, respectively). But the overall fertility rate of 26 live births in 106 women (0.25) was significantly lower than that in the general population (0.25 vs 1.8; *P* < .001).^[19]^

Previous report suggested that women with CAH who became pregnant might be at risk for GDM but it has not been proved.^[20]^ The patient in our case was diagnosed as GDM during the second trimester but her blood sugar was well-controlled by lifestyle modification. The rate of other complications such as preeclampsia or premature birth does not seem to increase.^[19]^ Cesarean section is usually conducted in individuals with a history of vaginoplasty,^[21]^ as did in our report. For women diagnosed with CAH who carry an healthy baby, prednisolone, hydrocortisone, or prednisone are optional steroids because these medications do not affect the fetus.^[21,22]^ The patient in the study continued to take prednisolone 10 mg/d during pregnancy, and her baby had shown normal external genitalia with normal karyotype and normal development up to 6 years old. However, there is no established guidelines or consensus on the management of glucocorticoid and/or mineralocorticoid doses during pregnancy.

## 4 Conclusion

In conclusion, some mild CAH patients with certain types can achieved successful pregnancy without any assisted reproductive technology after they are treated with steroid therapy. However, there is no established guidelines or consensus on the management of glucocorticoid and/or mineralocorticoid doses during pregnancy. The pregnancy rate among CAH women who wish to conceive may be much more optimistic than previous researches although large sample rather than case reports is needed for further observations.

## Author contributions

**Conceptualization**: Wei Xiong, Guiqiong Huang, Xiaodong Wang, Shiqiao Tan.

**Investigation**: Wei Xiong, Guiqiong Huang.

**Supervision**: Shiqiao Tan.

**Writing – original draft**: Wei Xiong, Guiqiong Huang.

**Writing – review & editing**: Xiaodong Wang, Shiqiao Tan.
